# Depletion of VAX2 Restrains the Malignant Progression of Papillary Thyroid Carcinoma by Modulating ERK Signaling Pathway

**DOI:** 10.1515/biol-2019-0027

**Published:** 2019-07-10

**Authors:** Bei Guo, Yi Zhang, Kun Yuan, Feng-Xia Jiang, Qian-Bo Cui, Qin Zhou, Hong-Xia Dong, Wei Chen, Shun-Shi Yang

**Affiliations:** 1 Department of Otorlaryngology Head and Neck Surgery, The Central Hospital of Wuhan, NO.26 Shengli street, Jiangan district, Wuhan, Hubei 430014, P.R. China; 2 Department of Ultrasound, The Central Hospital of Wuhan, NO.26 Shengli street, Jiangan district, Wuhan, Hubei 430014, P.R. China; 3 Department of Otorlaryngology Head and Neck Surgery, The Central Hospital of Wuhan, Tongji Medical College, Huazhong University of Science and Technology, Wuhan, Hubei, China; 4 Department of Ultrasound, The Central Hospital of Wuhan, Tongji Medical College, Huazhong University of Science and Technology, Wuhan, Hubei, China

**Keywords:** VAX2, TCGA, prognosis, proliferation, ERK signaling pathway, papillary thyroid carcinoma

## Abstract

**Objective:**

Ventral anterior homeobox 2 (VAX2) gene is a key regulating factor for the development of the ventral region of the eye, and has recently attracted much attention from the cancer treatment field. Our study aimed to explore the effect of VAX2 on papillary thyroid carcinoma (PTC).

**Methods:**

We determined the expression levels of VAX2 in PTC based on The Cancer Genome Atlas (TCGA) database. We then assessed the prognosis of patients with PTC, and analyzed the association between VAX2 expression and clinicopathological characteristics. Subsequently, we measured the biological functions of VAX2 in PTC using qRT-PCR, cell counting kit-8 (CCK-8) assay, colony formation assay, wound healing assay, transwell assays and western blot.

**Results:**

VAX2 was up-regulated in PTC tissues when compared with normal thyroid tissues, and high expression level of VAX2 was positively correlated with poor prognosis. Furthermore, knockdown of VAX2 significantly inhibited the proliferation, migration and invasion of PTC cells. Importantly, through western blot analysis, we found that the expression of phosphorylated-(p) ERK and p-MEK in ERK signaling pathway showed a significant decrease after knockdown of VAX2.

**Conclusion:**

These findings suggest that VAX2 may be involved in the malignant progression of PTC, and hold significant potential as a therapeutic target for PTC.

## 1 Introduction

Papillary thyroid carcinoma (PTC) is one of the most widespread types of endocrine malignancies, constituting approximately 80% of all thyroid carcinomas [1, 2, 3]. The incidence and morphology of PTC have been reported to be influenced by dietary iodine deficiency [4, 5]. Currently, high-resolution sonography is the primary tool for preoperative thyroid cancer evaluation, which can diagnose the variants of PTC, influence whether to perform a biopsy, and determine the extent of surgery, etc. [6, 7, 8]. After traditional surgical treatment and radioiodine ablation therapy, most PTC patients have excellent prognoses with very high survival rates [8, 9, 10, 11]. However, a high recurrence rate is observed after the traditional therapy and several patients still succumb to PTC [5, 8, 9, 12]. Moreover, there are no effective therapeutic methods currently available for those patients who are not eligible for surgery and develop radioiodine resistance [5]. Hence, to reduce the recurrence rate and the mortality of PTC, seeking an efficient treatment remains a challenge.

Ventral anterior homeobox 2 (VAX2) gene belongs to a subfamily of homeobox genes closely related to the *Drosophila empty spiracles* and to its vertebrate homologues, the Emx genes [13, 14]. VAX2 gene has been reported to be specifically expressed in the ventral region of the prospective neural retina in vertebrates [15, 16]. Accumulating evidences indicate that VAX2 plays crucial roles in regulating the proper development of the ventral region of eye [15, 16, 17, 18]. As a growing number of research focuses on VAX2, the roles of VAX2 on other diseases have been revealed. Norgett et al. have found that a novel genomic deletion at 2p13.3 encompassing all of ATP6V1B1 and part of VAX2 cause distal Renal Tubular Acidosis, which indicates that VAX2 may be associated with distal Renal Tubular Acidosis [19]. Moreover, Kitchen et al. analyzed the genome-wide methylation in high-grade non-muscle invasive bladder cancer and found that promoter methylation was significantly correlated with reduced transcript expression for VAX2, ARHGEF4, PON3 and STAT5a gene [20]. They demonstrated that VAX2 and other genes hold significant potential as targets for novel therapeutic intervention, with traditional therapeutic options in the treatment of bladder cancer [20]. Based on these findings, our objective was to determine potential effects of VAX2 on PTC.

In this study, we determined the expression level of VAX2 in PTC based on The Cancer Genome Atlas (TCGA) database. We then assessed the prognosis of patients with PTC, and analyzed the association between VAX2 expression and clinical characteristics. Subsequently, the biological functions of VAX2 in PTC were measured and the results indicated that VAX2 may be involved in the malignant progression of PTC.

## 2 Materials and Methods

### 2.1 Data source

The RNA-seq dataset, which consists of the RNA expression data from 510 PTC tissues and 58 normal thyroid tissues was obtained from TCGA database (https://cancergenome.nih.gov/) The corresponding full-scale clinical information of 446 PTC patients was also collected from TCGA database.

### 2.2 Cell culture and transfection assay

Nthy-ori3-1, BCPAP and TPC-1 cell lines were purchased from Shanghai Institutes for Biological Sciences (Shanghai, China). Cells were maintained in RPMI-1640 medium supplemented with 10% fetal bovine serum (FBS), 100 U/ml penicillin, and 0.1 mg/ml streptomycin. Cells were transfected with two types of siRNAs (si-VAX2 1: 5’-UUCGGGAAAUUGUCCUGC C-3’; si-VAX2 2: 5’-GCAGAAGAAAGACCAGAGC-3’). Non-specific siRNA was used as a negative control group (si-con). After transfection for 48 h, the transfected cells were collected for the subsequent experiments. Transfection efficiency was detected by quantitative real-time reverse transcription-PCR (qRT-PCR) and western blot.

### 2.3 RNA extraction and qRT-PCR

Total RNA was extracted using TRIzol extraction Kit (Invitrogen, Grand Island, NY, USA) according to the manufacturer’s instructions. cDNA was synthesized using Reverse Transcription Kit (Takara, Dalian, China). The RNA expression levels were measured by qRT-PCR using SYBR Green PCR Master Mix (Takara, Dalian, China), which was performed on the ABI 7500 Fast Real Time PCR system (Applied Biosystems, Foster City, CA, USA) as follows: 95℃ for 5 min, 40 cycles of 95℃ for 30 sec, 60℃ for 40 sec, and 72℃ for 1 min. GAPDH was used as an internal control. Comparative quantification was assessed using the 2^−ΔΔCt^ method. The primers are presented as follows: VAX2 forward, 5’-CCT TAG GTG ACC CCA GGA AC-3’, and reverse, 5’- CGA GGA ACC CAG TTC GTA GC-3’; GAPDH forward: 5’-GGA GCG AGA TCC CTC CAA AAT-3’ and reverse, 5’-GGC TGT TGT CAT ACT TCT CAT GG-3’.

### 2.4 Western blot

Total proteins were obtained from the cells by RIPA buffer (KeyGEN, Nanjing, China) with protease inhibitors. A BCA kit (KeyGEN, Nanjing, China) was used for protein quantification. Quantified protein lysates were separated on SDS-PAGE gels, transferred onto PVDF membranes, blocked with 5% skimmed milk for 1 h at room temperature, and incubated overnight at 4°C with primary antibodies (dilution 1:1000) against MEK (Cat no. 9126, Cell Signaling Technology, Inc., Danvers, MA, USA), phosphorylated-(p)-MEK (Cat no. 3958, Cell Signaling Technology, Inc., Danvers, MA, USA), ERK (Cat no. 4695, Cell Signaling Technology, Inc.), p-ERK (Cat no. 8544, Cell Signaling Technology, Inc.), GAPDH (Cat no. 5174, Cell Signaling Technology, Inc.) and VAX2 (Cat no. 15773-1-AP, Proteintech Group, Rosemont, Illinois, USA ). After incubation with the secondary antibody (Anti-rabbit IgG, HRP-conjugated goat anti-rabbit, dilution 1:3000, Cat no. 7074, Cell Signaling Technology, Inc.), the proteins were visualized using ECL detection system (Thermo Fisher Scientific, Inc., Waltham, MA, USA). The intensity of the signals on each membrane was quantified by densitometry (Quantity One software, Bio-Rad, Sunnyvale, CA, USA). GAPDH was used as internal control.

### 2.5 Cell Counting Kit-8 (CCK-8) assay

CCK-8 assay was used to determine the cell proliferation. After transfection for 24 h, cells were seeded on 96-well plates at a density of 1000 cells/well. Cell viability was determined every 24 h according to the manufacturer’s instructions. Optical density of the samples was measured at 450 nm using a microplate reader (Thermo Scientific Microplate Reader, Waltham, Massachusetts, USA).

### 2.6 Colony formation assay

The transfected cells were placed onto 6-well plates at the density of 1000 cells/well and maintained in medium with 10% FBS. The medium was replaced every 3 or 4 days. After incubation for two weeks, the colonies were fixed with 4% paraformaldehyde for 30 min and stained with 0.1% crystal violet for 30 min. The colonies were imaged and counted.

### 2.7 Wound healing assay

Cells were cultured in 6-well plates at a density of 5 × 10^5^ cells/well for 24 h, resulting in a monolayer at more than 90% confluence. A wound was created in a line across the plate by scraping with a 100 μl plastic pipette tip. The scratched monolayer was washed twice with PBS to remove all cell debris and then cultured with the RPMI 1640 medium for another 24 h. Photomicrographs were recorded at 0 h and 24 h by an Olympus BX51 microscope (Olympus Corporation, Tokyo, Japan).

### 2.8 Cell migration and invasion assays

Transwell experiments were performed to determine the migration and invasion of cells. For the migration assays, the transfected cells were seeded on the upper chamber of an insert (8 μm pore size, BD Biosciences, Lake Franklin, New Jersey, USA). For the invasion assays, the transfected cells were placed into the upper chamber coated with Matrigel (Sigma Aldrich, USA). The culture medium (500 μl) containing 10% FBS was added into the lower chamber. After incubation at 37℃ for 12 h, non-migrated cells that remained in the upper chambers were removed by cotton swabs. The cells that migrated to the underside of the membrane were fixed with paraformaldehyde for 30 min, stained with 0.1% crystal violet for 20 min, washed with PBS, counted and imaged under an Olympus BX51 microscope (Olympus Corp., Shinjuku, Tokyo, Japan).

### 2.9 Statistical analysis

In this study, all experiments were repeated at least three times. Data are expressed as mean ± standard deviation (SD), and were analyzed using SPSS Statistics software (version 22.0, Chicago, IL, USA) and GraphPad software (GraphPad Software, Inc., La Jolla, CA, USA). Significant differences were determined using Student’s T-test. One way ANOVA was used to evaluate the significant difference among multiple groups. The relationship between VAX2 expression levels and clinicopathological characteristics was calculated by Chi-square test. Samples were divided into high and low expression groups based on the median of the VAX2 expression. The median of VAX2 expression was 7.57. The overall survival (OS) rates were assessed using the Kaplan–Meier method with the log-rank test applied for comparison. Univariate and multivariate Cox proportional hazards analyses were used to evaluate the independent prognostic factors for survival in PTC patients. Statistical significance was determined as p < 0.05.

**Ethical approval**: The conducted research is not related to either human or animals use.

## 3 Results

### 3.1 VAX2 expression is up-regulated in PTC tissues

**Figure 1:**
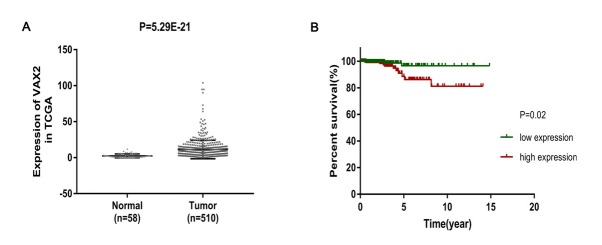
Expression level of ventral anterior homeobox 2 (VAX2) in papillary thyroid carcinoma (PTC) was markedly increased and positively associated with the prognosis of patients. (A) VAX2 expression levels of PTC tissues (n = 510) and normal thyroid tissues (n = 58) from The Cancer Genome Atlas (TCGA) database were evaluated. (B) The overall survival (OS) analysis for two groups of patients with low (n = 223) or high (n = 223) expression levels of VAX2 (p = 0.02). PTC patients were divided into low and high expression group based on the median expression of VAX2 (7.57).

To investigate the potential role of VAX2 in PTC, we first evaluated VAX2 expression in PTC based on the RNA-seq data from TCGA. The result showed that VAX2 expression was obviously up-regulated in PTC tissues compared with normal thyroid tissues (p < 0.05, Figure 1A)

We further analyzed the relationship between VAX2 expression level and clinicopathological characteristics using Chi-square test. As shown in table 1, VAX2 expression level was positively correlated with age (p < 0.001), clinical stage (p = 0.007), pathologic-T (0.011) and death (p = 0.03). However, there was no significant difference between VAX2 expression and other clinicopathological characteristics such as gender (p = 0.749), pathologic-N (p = 0.776) and pathologic-M (p = 1). These results suggest that VAX2 may be involved in the malignant progression of PTC.

**Table 1:** Correlation of clinicopathological characteristics and VAX2 expression in PTC patients.

Characteristics	Expression of VAX2		P value
	
	Low	High	
**Age**			0.000*
<60	193	147	
≥60	30	76	
**Gender**			0.749
female	161	164	
male	62	59	
**Clinical-Stage**			0.007*
I+II	159	132	
III+IV	64	91	
**Pathologic-T**			0.011*
T1+T2	147	120	
T3+T4	76	102	
**Pathologic-N**			0.776
N0	115	112	
N1	108	111	
**Pathologic-M**			1.000
M0	220	219	
M1	3	4	
**Death**			0.030*
Yes	3	11	
No	220	212	

PTC patients were divided into low and high expression group based on the median expression of VAX2. The median of the VAX2 expression was 7.57.

Subsequently, we evaluated the relationship between the VAX2 expression and the survival time of PTC patients using the Kaplan–Meier method and the log-rank test. The overall survival (OS) curves indicated that PTC patients with high VAX2 expression exhibited a reduced OS (p = 0.02, Figure 1B) In addition, univariate analysis of prognostic variables showed that VAX2 expression (p = 0.032), clinical-stage (p = 0.001), pathologic-T (p = 0.048), and age (p < 0.001) were significantly related to OS in PTC patients (table 2). Multivariate analysis revealed that age was an independent prognostic factor for PTC patients (p = 0.002) but VAX2 expression cannot be used as an independent prognostic factor for PTC patients (p = 0.747). These findings suggest that high expression of VAX2 can predicate a poor prognosis of some PTC patients.

**Table 2:** Univariate and multivariate analysis of prognostic parameters in PTC patients using the COX regression model.

Variables	Univariate analysis			Multivariate analysis		
	
	P	HR	95%CI	P	HR	95%CI
VAX2 expression（high/low）	0.032	4.033	1.124-14.463	0.747	1.250	0.322-4.852
Clinical-Stage（I+II/III+IV）	0.001	8.258	2.298-29.677	0.682	1.416	0.268-7.488
Pathologic-T (T1+T2/T3+T4)	0.048	3.228	1.010-10.315	0.783	1.225	0.288-5.221
Pathologic-M (M0/M1)	0.387	2.467	0.319-19.052			
Pathologic-N (N0/N1)	0.722	1.213	0.420-3.503			
Age(＜60/≥60）	0.000	47.422	6.194-363.044	0.002	33.861	3.575-320.707
Gender (female/male)	0.308	1.770	0.590-5.304			

### 3.2 VAX2 expression is up-regulated in PTC cells

**Figure 2:**
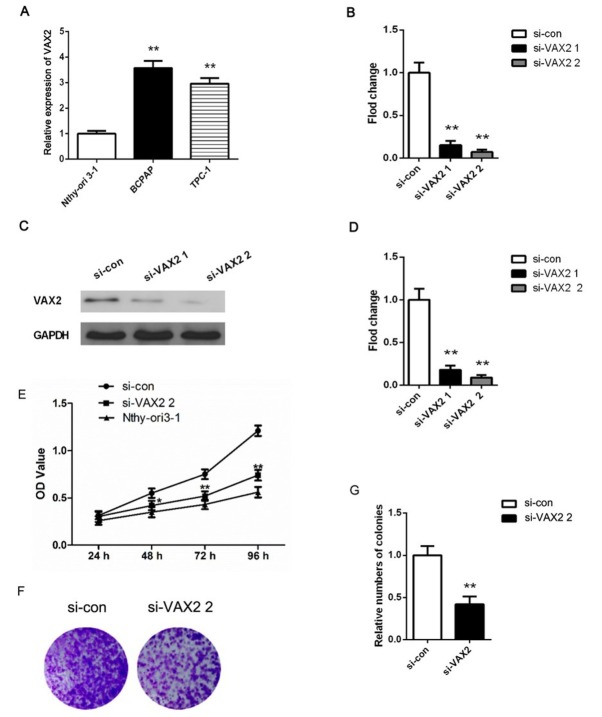
(A) Expression level of VAX2 in PTC cells was significantly increased. The expression levels of VAX2 in PTC cell lines (BCPAP and TPC-1) and normal thyroid cell line (Nthy-ori3-1) was measured by qRT-PCR. BCPAP cell line was selected as an appropriate cellular model for the following experiments. (B) qRT-PCR and western blot (C, D) were used to determine VAX2 expression in BCPAP cells after transfection. We used two types of siRNAs (si-VAX2 1 and si-VAX2 2) to silence the VAX2 expression. si-VAX2 2 was selected to knock down the VAX2 gene for the further study. (E) The proliferation of BCPAP and Nthy-ori3-1 cells was assessed by CCK-8 assay. (F-G) Colony formation assay further determined the proliferation of BCPAP cells. *p < 0.05

To investigate the biological function of VAX2 in PTC cells, we first used qRT-PCR to measure VAX2 expression in PTC cell lines (BCPAP and TPC-1) and normal thyroid cell line (Nthy-ori3-1). As compared to Nthy-ori3-1 cell line, the expression levels of VAX2 were up-regulated in PTC cell lines, especially BCPAP cell line (Figure 2A p < 0.05). Thus, we chose BCPAP cell line as an appropriate cellular model for the following experiments.

Furthermore, we used two types of siRNAs (si-VAX2 1 and si-VAX2 2) to silence the VAX2 expression. In qRT-PCR assay, the mRNA expression levels of VAX2 were considerably decreased in BCPAP cells after transfected with si-VAX2 1 or si-VAX2 2 (Figure 2B p<0.01). Western blot further confirmed the result of qRT-PCR assay (Figure 2C, 2D) The transfection efficiency in si-VAX2 2 group was relatively high than that in si-VAX2 1 group. Thus, si-VAX2 2 was selected to knock down the VAX2 gene for the further work.

### 3.3 Effect of VAX2 expression on PTC cell proliferation

To investigate the effect of VAX2 expression on PTC cell proliferation, CCK-8 and colony formation assays were used to determine the viability of BCPAP cells. CCK-8 assays demonstrated that knockdown of VAX2 markedly reduced the cell proliferation at 48 h (p < 0.05), 72 h and 96 h (p<0.001, Figure 2E) compared with the si-con group. The proliferation ability of Nthy-ori3-1 cells was significantly weaker than BCPAP cells (p<0.01, Figure 2E) From Figure 2F and 2G we observed that colony formation ability was greatly decreased after knockdown of VAX2 in BCPAP cells (p < 0.01). These results indicate that VAX2 has an oncogenic effect to promote the proliferation of BCPAP cells.

### 3.4 Effect of VAX2 expression on PTC cell migration and invasion

**Figure 3:**
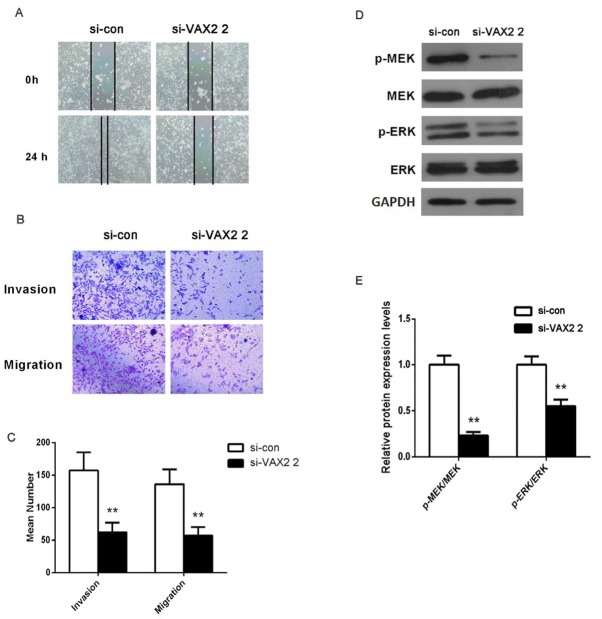
(A) The migration and invasion of the transfected cells were measured by wound healing assay (X100). (B, C) Subsequently, the migration and invasion of the transfected cells were evaluated by transwell assays (X200). (D, E) The effect of VAX2 expression on ERK signaling pathway was determined by western blot. GAPDH was used as internal control. *p < 0.05

To investigate the effect of VAX2 expression on PTC cell mobility, wound healing assay and transwell assay were performed to assess the migration and invasion of BCPAP cells. As shown in Figure 3A knockdown of VAX2 obviously inhibited the migration of BCPAP cells because the width of the wound in si-VAX2 2 group was longer

compared with si-con group. In transwell assays, we observed that knockdown of VAX2 significantly decreased the migrated and invaded cell numbers in si-VAX2 2 group compared with si-con group (p < 0.01, Figure 3B and 3C) These data suggest that the motility of PTC cells was remarkably reduced after silencing VAX2.

### 3.5 Effect of VAX2 expression on ERK signaling pathway

To explore a potential mechanism for VAX2 in the malignant progression of PTC, we measured the expression levels of key proteins in the ERK signaling pathway. Western blot demonstrated knockdown of VAX2 led to an obvious decrease in the expression levels of p-MEK and p-ERK as compared to si-con group (Figure 3D and 3E p < 0.05). Meanwhile, the expression levels of MEK and ERK had no significant differences between si-con group and si-VAX2 2 group. The results suggest that knockdown of VAX2 markedly inhibit the activation of ERK signaling pathway.

## 4 Discussion

The results of bioinformatics analysis and biological experiments indicate that VAX2 is up-regulated in PTC and is associated with poor prognoses. Meanwhile, knockdown of VAX2 expression significantly inhibited the proliferation, migration and invasion of BCPAP cells, which suggest that the high expression levels of VAX2 could promote the growth of BCPAP cells. Moreover, knockdown of VAX2 suppressed the activation of the ERK signaling pathway, which might be involved in regulating the proliferation, migration and invasion of BCPAP cells. Thus, VAX2 may be a potential therapeutic target for PTC.

According to the previous research, VAX2 gene is specifically expressed in the ventral region of the prospective neural retina in vertebrates, which plays an important role in regulating the proper development of the ventral region of eye [15-18]. In addition, it is reported that VAX2 is involved in the pathological process of other diseases such as distal Renal Tubular Acidosis [19] and high-grade non-muscle invasive bladder cancer [20]. The genome-wide methylation analysis in high-grade non-muscle invasive bladder cancer revealed that promoter methylation was positively correlated with reduced transcript expression for VAX2 , ARHGEF4, PON3 and STAT5a gene, which suggested that VAX2 and other genes could be used as novel therapeutic targets for bladder cancer [20]. Inspired by these finding, we investigated the effects of VAX2 on PTC. Based on the TCGA database, we used the bioinformatics methods to analyze VAX2 expression in PTC. The results indicated that VAX2 was up-regulated in PTC tissues compared with normal thyroid tissues and VAX2 expression was positively associated with poor prognosis. We further assessed the biological functions of VAX2 in PTC cells. As a result, depletion of VAX2 limited the proliferation, migration and invasion of BCPAP cells. Accordingly, it is likely that VAX2 shows significant potential as a therapeutic target for PTC.

ERK signaling pathway, as a central MAPK pathway, regulates various cellular processes mainly associated with differentiation, migration, invasion, proliferation, etc. [21, 22, 23]. When one of the proteins such as ERK and MEK in the pathway is mutated, it can become stuck in the “on” or “off” position, which is a necessary process in the progression of many cancers [24, 25]. In tumors, including ovarian carcinoma, cervical neoplasms, prostate cancer, and PTC etc., the levels of p-MEK and p-ERK are often markedly higher than that in the normal tissues[26, 27, 28, 29]. ERK signaling pathway has been demonstrated to be one of most frequently dis-regulated pathways in PTC. Sprecht et al. reported that 6 of 10 samples of surgically resected PTCs exhibited enhanced expression of p-ERK in tumor tissues compared with the adjacent normal tissues [29]. The Hong group found that miR-20b displays tumor-suppressor functions in PTC by regulating the ERK signaling pathway [30]. The Cheng group also found that ERK signaling pathway plays critical roles in the progression of PTC [31]. Hence, we determined the situation of ERK signaling pathway in PTC cells. The results showed that the expression levels of p-ERK and p-MEK were obviously decreased after knockdown of VAX2. Therefore, these findings suggested that depletion of VAX2 curbed the proliferation, migration and invasion of PTC cells might occur by altering the ERK signaling pathway. However, the specific mechanisms by which VAX2 affects the ERK signaling pathway still remains unknown and is an area for future research.

In conclusion, our study found that VAX2 was up-regulated in the PTC tissues and cells and positively associated with poor prognosis in PTC patients. Moreover, silencing VAX2 repressed the growth and motility of PTC cells, which might be through inhibiting the ERK signaling pathway. Hence, VAX2 may be a potential therapeutic target for PTC. In future studies, we will consider broadening the scope of the assays, and potentially include animal experiments as well as clinical trials in order to further investigate the findings presented here.
